# Reduced Graphene Oxide Inserted into PEDOT:PSS Layer to Enhance the Electrical Behaviour of Light-Emitting Diodes

**DOI:** 10.3390/nano11030645

**Published:** 2021-03-05

**Authors:** Fernando Rodríguez-Mas, Juan Carlos Ferrer, José Luis Alonso, Susana Fernández de Ávila, David Valiente

**Affiliations:** Communications Engineering Department, Universidad Miguel Hernández, 03202 Elche, Spain; jc.ferrer@umh.es (J.C.F.); j.l.alonso@umh.es (J.L.A.); s.fdezavila@umh.es (S.F.d.Á.); dvaliente@umh.es (D.V.)

**Keywords:** cadmium sulphide, PVK, hybrid light-emitting device, electroluminescence, nanocrystals, reduced graphene oxide

## Abstract

In this study, poly(9-vinylcarbazole) (PVK)-based LEDs doped with reduced graphene oxide (rGO) and cadmium sulphide (CdS) nanocrystals were fabricated by spin-coating. The hybrid LED structure was a layer sequence of glass/indium tin oxide (ITO)/PEDOT:PSS|rGO/PVK/Al. rGO was included in the poly(3,4-ethylenedioxythiophene)-poly(styrenesulfonate) (PEDOT:PSS) layer due to its energy bands being close to PEDOT:PSS bands, and the possibility of using water for dispersing both polymer and flakes. Optical properties such as photoluminescence and UV-Vis absorption were not affected by the addition of rGO to the PEDOT:PSS solution. However, PVK-based LEDs with rGO showed increased current density compared to those without rGO in the hole transporting layer. Higher electroluminescence intensities were observed for rGO-enriched LEDs, although the shape of the spectrum was not modified. LEDs including CdS nanocrystals in the poly(9-vinylcarbazole) emissive layer did not show such dependence on the rGO presence. Though the addition of rGO to PEDOT:PSS still produces a slightly higher current density in CdS doped LEDs, this growth is no longer proportional to the rGO load.

## 1. Introduction

Organic-semiconductor devices have gained popularity in recent years due to several advantages they offer compared to their inorganic counterparts, namely mechanical flexibility, low cost [1], and a simple manufacturing processes [2,3]. Deposition of organic polymer layers can be achieved by means of simple techniques such as spin-coating. With this technique, uniform layers are applied onto a flexible or rigid substrate by means of centrifugal force. In these devices, specifically in light-emitting diodes (LEDs), the optical and electrical properties could be modified by the inclusion of different dopants, such as graphene and its derivatives, or semiconductor nanocrystals.

Since 2004 [4], graphene and its derivatives have generated high expectations in several areas, including as solar cells [5], field-effect transistors [6], electrocatalyst [7], transparent electrodes [8], graphene transistors [9], etc. Graphene has excellent properties, such as excellent thermal conductivity [10], electron mobility [11] and transparency [12], that make possible its involvement in the aforementioned areas. The simplest process for synthesizing graphene consists in the oxidation and exfoliation of graphite. This process transforms graphite into graphene oxide (GO). In a later step, GO is reduced, and either graphene or reduced graphene oxide (rGO) is synthesized depending on the reduction degree.

Not all the properties of graphene are extensible to graphene derivatives (GO or rGO). In the case of reduced graphene oxide, certain properties are maintained, but to a lesser extent, such as a conductivity of 200 S/m^1^. In this paper, we will study the possibility of manufacturing hybrid LEDs with graphene derivatives. Normally, in these devices, the hole transport layer is formed by Poly(3,4-ethylenedioxythiophene)-poly(styrenesulfonate) (PEDOT:PSS), but in our analysis, this layer will be doped with different proportions of rGO. Reduced graphene oxide was chosen because the value of its energy bands (−5.2~−4.9 eV) [13] is very close to PEDOT:PSS, (−5.1~−5 eV) [13]. Additionally, rGO is aqueous dispersible, like the PEDOT:PSS, eliminating the possible problems that a mixture of different solvents might cause in the spin-coating technique [14].

On the other hand, Cadmium sulphide (CdS) nanocrystals (NCs) have been studied in recent years [15] due to their ability to modify the optical and electrical properties of the organic devices [16]. Cadmium sulphide nanoparticles have been used to improve or modify the properties of different devices, such as organic solar cells [17], light-emitting devices [18], hybrid memory devices as a compound of polymer composites and CdS nanocrystals [19,20], and memory devices with other nanoparticles, such as graphene oxide nanocrystals [21]. CdS is a semiconductor with a direct bandgap energy of 2.4 eV. This bandgap energy makes it suitable for applications in the visible band of the electromagnetic spectrum. A simple way to synthesize CdS nanocrystals could be through the process of thiolate decomposition [22]. This route is based on a two-step process: (i) synthesis of a thiolate (compound formed by the future surface ligand and the precursor metal of the nanoparticle) and (ii) reaction with a sulphur source, originating the nanocrystals. It should not be overlooked that a critical factor of nanocrystal behaviour is their size [23,24], since the optical properties depend on this parameter. To control the size and solubility, the nanocrystals are coated with ligands [25]. In this route, the nanocrystals are coated with thiophenol. The presence of thiophenol makes them soluble in dimethyl sulfoxide (DMSO). However, since the control of the exact mass of nanoparticles in solution is imprecise with this method, they were dried following reference [14]. Moreover, thiophenol was chosen as a ligand because thiophenol is formed by an aromatic ring [26]. This aromatic ring is expected to improve the charge transfer between the nanocrystals and the surrounding polymer because of the ring resonance. The influence of rGO on devices with their emission layer doped with CdS was also studied.

The aim of this paper is to analyse the influence of rGO and CdS nanoparticles embedded in PVK polymer on the electrical and optical properties of hybrid LEDs with active layers based on these materials.

## 2. Materials and Methods

### 2.1. Materials

Cadmium nitrate-tetrahydrate (Cd(NO_3_)_2_·4H_2_O, 99.99%), thiophenol (99%), sulphur powder (99.98%), poly(9-vinylcarbazole) (PVK, 98%), poly(3,4-ethylenedi-oxythiophene):poly(styrene sulfonate) (PEDOT:PSS, 1.3% water solution), toluene, methanol and dimethyl sulfoxide (DMSO) were purchased from Sigma-Aldrich (Darmstadt, Germany) and used without further purification. Reduced graphene oxide (rGO) was obtained from Graphenano “nanotechnologies” (Yecla, Spain) and used without further purification.

### 2.2. Characterization

The measurements of optical absorption were carried out with a T92+ UV/VIS spectrophotometer from PG instruments Ltd. (Lutterworth, UK), and the measurements of photoluminescence (PL) were performed with a Modular Spectrofluorometer Fluorolog-3 from Horiba Scientific (Madrid, Spain). In all the photoluminescence measurements, the excitation wavelength was fixed at λ_exc_ = 365 nm.

Transmission electron microscopy (TEM) analysis was performed using a Jeol 2010 (Tokyo, Japan) operating at 200 kV. High-resolution TEM (HRTEM) images were obtained by phase contrast at Scherzer defocus in order to obtain easily interpretable images.

Current density vs. voltage (J–V) curves of the LEDs were measured using Keithley 2400 Sourcemeter equipment (Bracknell, UK).

Electroluminescence (EL) characterization was performed with a Triax 190 monochromator (Madrid, Spain) and a multichannel thermoelectrically cooled CCD Symphony detector by Horiba Jobin Yvon (Madrid, Spain).

### 2.3. Synthetic Pathway of PEDOT:PSS|rGo Dispersions

A rGO solution was prepared and mixed with PEDOT:PSS. A reduced graphene oxide flake solution was prepared with distilled water at 4 wt%. When the solution was completely dispersed, it was mixed with the PEDOT:PSS, 1.3% water solution, in accordance with the proportions in volume shown in Table 1. The rGO was redispersed in distilled water because PEDOT:PSS was dissolved in water, too. In this sense, possible problems resulting from the phase separation of different solvents were avoided. In the spin-coating process, the use of solution with different solvents could cause craters and agglomerations in the deposited layer, as we observed in our previous study [14].

### 2.4. Synthesis of CdS Nanocrystals Powder

The route used for the synthesis of CdS NCs was an extension of the thiolate decomposition method [14]. The main advantage of this method is that nanocrystals end up in a powder, making it simpler to embed them in the organic polymer. The first step in this process was the synthesis of cadmium thiolate, Cd(C_6_H_5_S)_2_. For this, 1.54 g of Cd(NO_3_)_2_·4H_2_O was dissolved in 25 mL of distilled water and 25 mL of methanol and the solution was stirred for 30 min. In another flask, 1.03 mL of thiophenol was dissolved in 50 mL of distilled water. After that, when both solutions were well dissolved, the solutions were mixed and the blend was stirred. When Reaction (1) evolved, the mixture turned a whitish colour and the cadmium thiolate precipitated. The white powder was filtered and dried obtaining the cadmium thiolate.

Cd(NO_3_)_2_·4H_2_O + C_6_H_5_-SH → Cd(C_6_H_5_S)_2_↓ (1)

In a second step, two more solutions were prepared, 0.08 g of Cd(C_6_H_5_S)_2_ was dissolved in 2 mL of DMSO and, in another vial, 0.17 g of sulphur was dissolved in 20 mL of toluene. Both solutions were stirred for thirty minutes and when the thiolate was dissolved, 0.4 mL of sulphur solution was added. The mixture changed and turned yellowish, indicating that the CdS NCs had been synthesized correctly. The nanocrystals ended up dissolved in DMSO and it was necessary to eliminate the solvent in order to avoid phase separation when mixed with the polymer solvent. To achieve this, the yellowish solution was heated to 200 °C for one hour. Once the solvent was evaporated, CdS NCs were obtained.

### 2.5. Hybrid LEDs Fabrication

Hybrid light-emitting diodes with a layer sequence of ITO/PEDOT:PSS|rGO/PVK:CdS/Al were fabricated by spin-coating. Commercial glass substrates covered with a semitransparent ITO layer were routinely cleaned by sequential sonication in 1,2,4-trichlorobenzene, acetone and isopropyl alcohol, and then dried with N_2_.

Aqueous PEDOT-PSS dispersion with rGO was spin-coated onto the clean ITO surface and then annealed at 100 °C for 60 min. Then, the active layers were spin-coated and dried at 80 °C for 60 min.

Finally, the metallization of the cathodes was performed by evaporating aluminium in a high-vacuum chamber (10^−6^ mbar) until a thickness of 200 nm was achieved.

## 3. Results

### 3.1. CdS NCs Characterization

**Figure 1:**
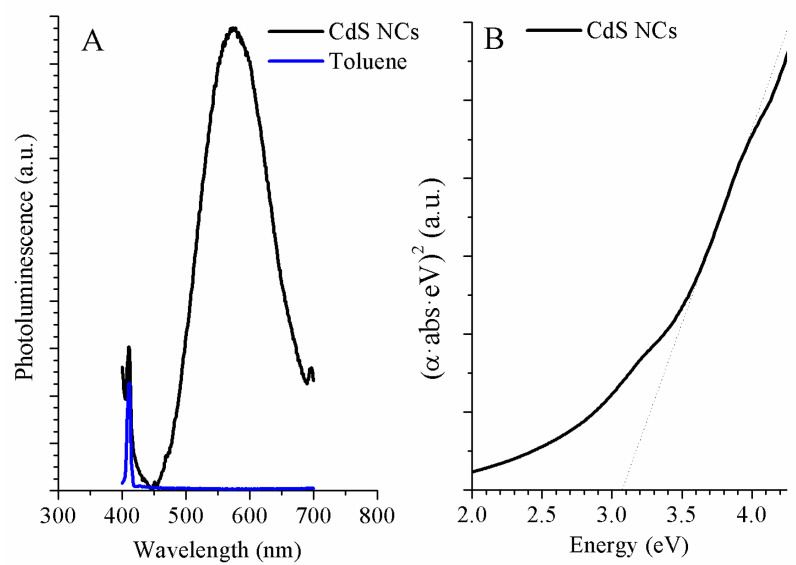
Photoluminescence spectra (**A**) of CdS nanocrystals (black line) and toluene (blue line). Optical absorption spectrum (**B**) of CdS NCs (black line).

Absorbance and photoluminescence measurements were performed. The PL spectra are shown in Figure 1A. Therein, a narrow peak is observed at low wavelengths, the maximum of the narrow peak had a wavelength at 411 nm. This peak corresponded with the maximum photoluminescence peak of toluene (blue line). The solvent peaks are not usually observed in PL graphs, because the emission intensity of nanoparticles normally hides the emission intensity of solvents. In this case, toluene was present in the CdS curve because the concentration of measured solution was very low. This low concentration made the emission intensity of nanoparticles lower. The low concentration can also explain the noise that was observed in the CdS photoluminescence. In this PL spectrum, the main peak shows its maximum at 576 nm.

To correctly observe the absorption edge and calculate the bandgap energy, the Tauc relation [27] was applied. In Figure 1B, (αhν)^2^ versus hν are plotted, showing an excitonic shoulder, pointing to the absorption edge (dotted line). The resulting value of the band gap is 3.07 eV (403.7 nm). According to this, the wavelength corresponding to the maximum intensity at the CdS NCs photoluminescence spectrum (Figure 1A) should be at lower wavelengths, close to the absorption edge. This shift towards higher wavelengths is due to defects in the nanocrystal surface. The defects originate deep surface states, producing trap emission.

To characterize the size of the nanoparticles, two different methods were used, a theoretical method, whose calculations are based on the band gap energy, and the direct measurement from TEM images. As a theoretical method, the equation suggested by Brus was employed [28].

*E_n_* = *E_b_* + (*ħ*^2^π^2^/2*R*^2^) × (1/m_e_* + 1/m_h_*) − 1.8e^2^/(4πε_0_ε*R*), (2)

where *E_n_* is the nanoparticles band gap, *E_b_* is the energy gap of the bulk material, m_e_* and m_h_* are the effective masses of electrons and holes, and ε is the dielectric constant. Solving the Brus equation (Equation (2)), these nanoparticles had an average size of 3.11 nm.

**Figure 2:**
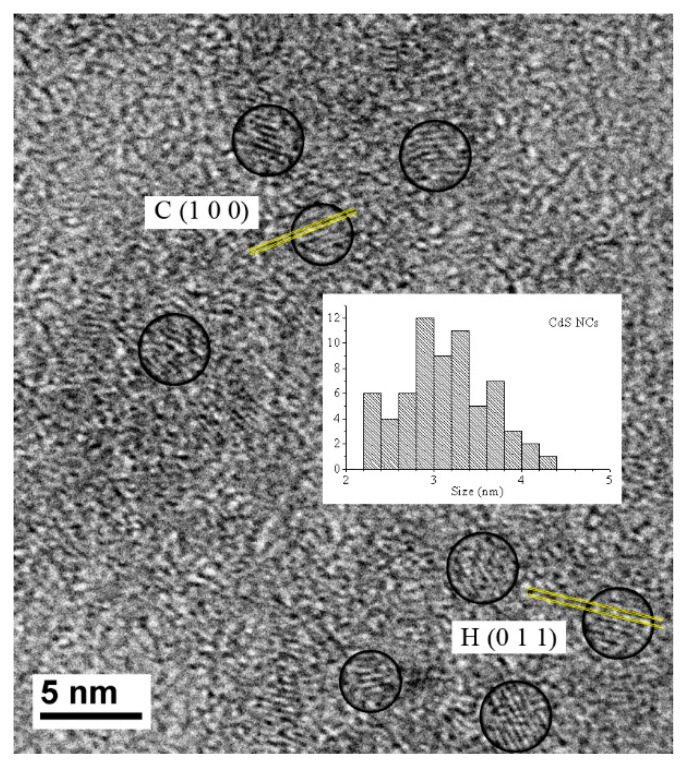
TEM images corresponding to CdS NCs and histogram of the size distribution for the nanocrystals.

To prepare samples for TEM analysis, a drop of CdS NCs solution in toluene was deposited on a carbon grid and then dried at room temperature. A high-resolution image of the CdS nanocrystals is shown in Figure 2, as well as the histogram of the size distribution of more than 50 particles. Diameters range from 2.2 nm to 4.4 nm, and the average size is 3.20 ± 0.06 nm. When comparing the size calculated from the Brus equation (Table 2) to the image measurement, a minimal difference can be observed (0.09 nm) that can be neglected. It was verified that the Brus equation performs a good approximation of the real size.

When measuring the interplanar distances of the CdS crystals in the HRTEM images, two different types of planes arise. The planes match the distances of the cubic zinc-blende-type structure and the hexagonal wurzite-type structure. In Figure 2, the {100} family planes of the cubic structure and the {011} family planes of the hexagonal phase are identified in two particles. The presence of both crystalline structures can justify the dispersion in the histogram of Figure 2. In addition, in Figure 1B, a second shoulder can be located at lower energy values. The dispersion in the histogram and the presence of the two shoulders in UV-Vis absorption, Figure 1B, confirm the two different structures [14]. The heat used to evaporate the solvent changes the NC structure. The energy could be enough to increase the size and transform the nanoparticles with cubic zinc blend structures into hexagonal wurzite-type structures [14,29,30].

### 3.2. Synthesis and Characterization of the Hybrid Solution

**Figure 3:**
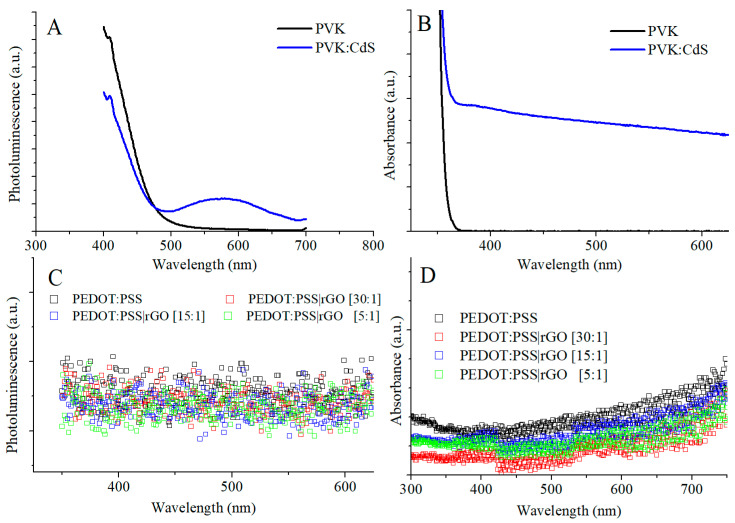
Photoluminescence (**A**) and absorption spectra (**B**) from pristine PVK and PVK doped with CdS NCs and photoluminescence (**C**) and absorption spectra (**D**) from PEDOT:PSS doped with different proportions of reduced graphene oxide.

As discussed in the introduction, the active layer of the devices was doped with CdS NCs. The active layer was prepared from a solution of PVK mixed with CdS nanocrystals. CdS nanoparticle powder was weighed and added to a solution formed by PVK dissolved in toluene. This solution had a PVK:CdS mass ratio of 8:1 at 3 wt%. Moreover, another solution with pristine PVK was also prepared with a concentration of 3 wt%. Optical absorption and photoluminescence measurements of these solutions were performed with the results shown in Figure 3.

The PL curves of pristine PVK and PVK with CdS NCs are plotted in Figure 3A. Both spectra exhibited a main peak corresponding to the PVK polymer located around 385 nm not shown in the figure. The influence of the nanoparticles was clearly visible in the blue line. The maximum of the secondary peak of the PVK solution with CdS NCs was localized at 577 nm. The same wavelength value that was presented in Figure 1A, where the CdS nanocrystals were measured without PVK. The photoluminescence of hybrid PVK shows a normal decrease in the PVK emission because the content of PVK polymer was reduced in the hybrid solution. Additionally, PVK photoluminescence quenching was observed [31]. In the hybrid solution, the excited electrons of the highest molecular orbital (HOMO) of the PVK polymer can either drift to the polymer’s lowest unoccupied molecular orbital (LUMO) or go to the conduction band of CdS. This migration produces a charge transfer, lowering the PVK intensity [13]. As for the optical absorption, a constant background absorption was observed in the total range of wavelengths (Figure 3B) due to the high concentration of CdS NCs.

To study the influence of reduced graphene oxide on the devices, rGO was included in several hybrid LEDs with different proportions. For this purpose, the hole transport layer, represented by the PEDOT:PSS polymer, was doped with different rGO masses.

We performed optical absorption and photoluminescence measurements to check the influence of rGO in our devices. To observe if the addition of reduced graphene oxide had any influence on the optical properties of the hole transport layer solution, absorbance and PL measurements were performed on the samples described in Table 1. The excitation wavelength for the photoluminescence measurements was λ_exc_ = 365 nm. To perform UV-Vis absorption measurements, PEDOT:PSS and PEDOT:PSS doped with rGO solutions were diluted to prevent re-absorption. Diluted solutions were measured.

No difference was observed between the PL curves of PEDOT:PSS doped with rGO and those of PEDOT:PSS, as shown in Figure 3C. There was no significant difference in optical absorption measurements, either (Figure 3D). Since the introduction of reduced graphene oxide on PEDOT:PSS did not influence the optical characteristics of PEDOT:PSS, we assume that it should not influence the optical characteristics of hybrid LEDs.

### 3.3. Hybrid LEDs with rGO

**Figure 4:**
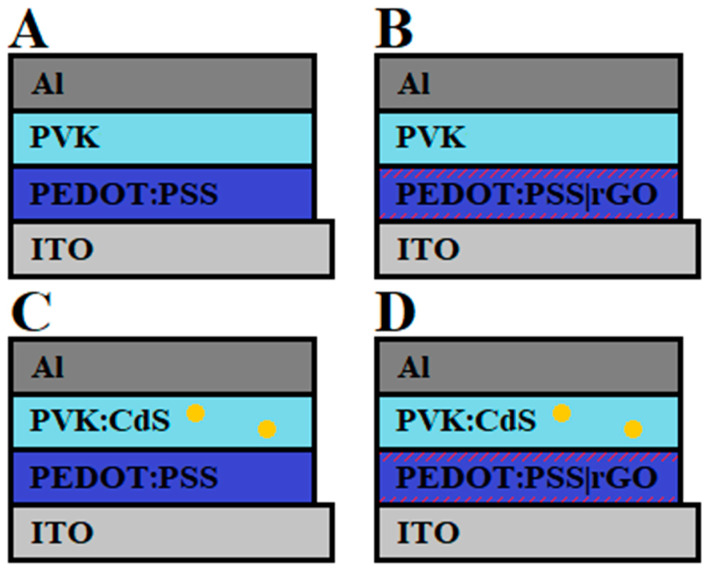
Structure of the different devices fabricated. ITO/PEDOT:PSS/PVK/Al (**A**), ITO/PEDOT:PSS|rGO/PVK/Al (**B**), ITO/PEDOT:PSS/PVK:CdS/Al (**C**), and ITO/PEDOT:PSS|rGO/PVK:CdS/Al (**D**).

Once the CdS nanocrystals and the different solutions were synthesized and characterized, hybrid light-emitting diodes were manufactured to check the influence of rGO on the devices. The hybrid LEDs consisted of a structure formed by the following stacked layers based on the solutions presented in the previous sections: glass/ITO/PEDOT: PSS(|rGO)/PVK(:CdS)/Al; Figure 4. Different devices were manufactured, employing PEDOT:PSS as a hole transport layer and PVK as the active layer. Excluding the changes in the concentrations of graphene and CdS nanoparticles, the same conditions were maintained for the fabricating process of LEDs.

Polymer and hybrid layers were deposited using the spin-coating technique. The hole transport layers were spin-coated at 2000 rpm on the substrate. When the hole transport layers were dried, the emission layers were deposited at 4000 rpm by spin-coating.

**Figure 5:**
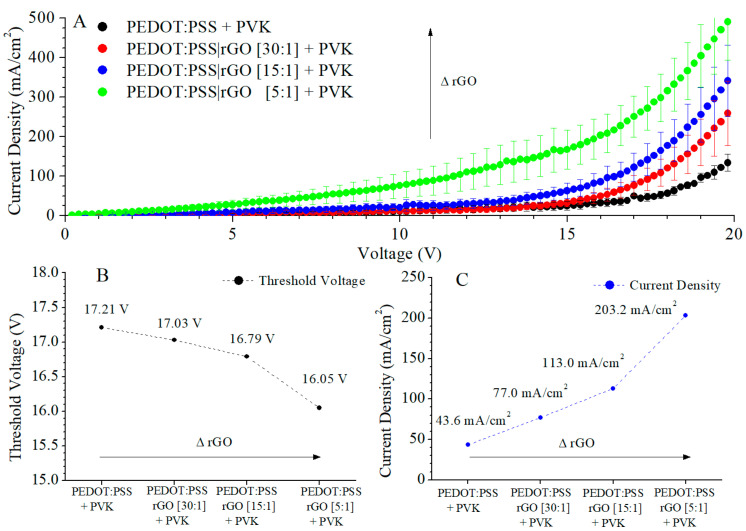
J-V curves (**A**), threshold voltage (**B**), and current density (**C**) at threshold voltage for the hybrid LEDs with rGO in the PEDOT:PSS layer.

Two types of hybrid LEDs were produced, without or with CdS NCs embedded in the PVK active layer. In the first one, the solutions indicated in Table 1 were employed to spin cast the hole transport layers, and pristine PVK was used as the emissive layer. An LED without reduced graphene oxide was manufactured as a reference. The emission layer was composed of PVK at 3% wt. When the devices were fabricated, we collected the measurements of current density vs. voltage (J-V). The curves are shown in Figure 5A.

Hybrid LEDs doped with rGO exhibited changes in electrical properties. The current density increases with the inclusion of rGO. The maximum improvement was found for the hybrid LED with [PEDOT:PSS|rGO] ≡ [5:1], and this decreases with the ratio of rGO. The current density values at the threshold voltage are shown in Figure 5C. This current increases with rGO concentration. On the other hand, the threshold voltage is reduced by the presence of rGO in the PEDOT:PSS layer.

The variation of the electrical behaviour in the hybrid LEDs indicated that the rGO inclusion in the PEDOT:PSS layer improved the hole transport [32]. rGO is a two-dimensional structure with graphene distributions. In graphene distributions, the charge carriers tend to move. The reduction by means of which graphene transforms into reduced graphene oxide eliminates part of the graphene conducting regions present in rGO [33]. In these distributions, the π-bond (C=C) and σ-bond (C-H) alternate, generating optimal paths for the hole transport. With the increase of reduced graphene oxide, the number of graphene distributions increases in the layer, augmenting the optimal paths for transport.

Next, we studied the electroluminescence of the hybrid LEDs doped with rGO. Regarding the emission intensity, LEDs doped with reduced graphene oxide showed higher emission intensity than reference PVK-LEDs, as indicated in [34,35].

**Figure 6:**
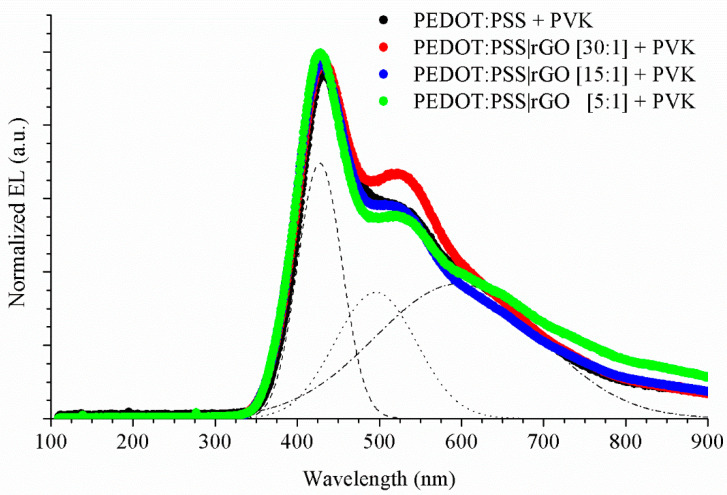
Normalized electroluminescence of PVK LED and hybrid LEDs using rGO in their hole transport layer. PEDOT:PSS + PVK (black), PEDOT:PSS|rGO [30:1] + PVK (red), PEDOT:PSS|rGO [15:1] + PVK (blue) and PEDOT:PSS|rGO [5:1] + PVK (green). Gaussian deconvolution of PEDOT:PSS + PVK (non-solid lines).

In Figure 6, the spectra were normalized to the maximum peak emission. All EL curves showed a similar morphology, with a narrow uppermost peak and two shoulders at longer wavelengths. To study these features, Gaussian deconvolutions were carried out for all spectra, indicated as non-solid lines in Figure 6 for PEDOT: PSS + PVK. Gaussian deconvolution is a statistical process where electroluminescence is decomposed by Gaussian curves, where the mean of each Gaussian curve coincides with the wavelength of the maximum emission peak, and the standard deviation is the difference of the wavelength of the maximum emission peak (mean) and the wavelength at which a $\left(1-1/\sqrt{e}\right)\%$ decrease from the maximum emission peak is produced. We allowed three Gaussian curves to fit each spectrum. The wavelengths corresponding to the maximum of each Gaussian emission peak are detailed in Table 3.

The EL spectra are different from the PL curves (Figure 3A). Like any polymer, PVK is a chain of monomers (a small molecule) bonded together. It is also known that photoluminescence of PVK has two peaks, one around 390 nm, produced by a small superposition of monomers, called p-PVK, because it is related to the phosphorescence of PVK. Another contribution is located around 410 nm, and is produced by the total superposition of the monomers, known as f-PVK, due to its relation to fluorescence of PVK [36]. In the EL curve of the PVK-reference, the peaks are not in the same positions as those observed for photoluminescence. The PVK electroluminescence presents a shift towards higher wavelengths. According to Ye et al. [36], with the increase of temperature, the emission intensity of p-PVK decreases and the emission intensity of the f-PVK increases and, at low temperatures, the PVK photoluminescence exhibits a broad peak at high wavelengths (~550 nm). This peak corresponds to the radiative transition of triplet states. In addition, Ye affirms that the PVK is a polar polymer, and that an electric current polarizes PVK. The polarization of PVK enhances the effect of f-PVK, since it reduces the intermolecular distance, and hence, the energy. Therefore, the peak of f-PVK is shifted towards higher wavelengths.

In PVK-reference, the maximum emission occurs for the narrowest peak, located at 428 nm, and the two shoulders that showed the EL curve were at 495 and 595 nm. The peak at 428 nm corresponds to the peak f-PVK enhanced by the electric field at room temperature. The two shoulders did not have any correspondence with the photoluminescence. Ye [36] stated that, for EL processes, the proportion of triplet states is increased compared to PL processes; this enables the observation of phosphorescent emissions at room temperature. This is the origin of the peak located at 495 nm. Finally, the lower shoulder observed in the EL spectrum close to a wavelength of 600 nm is attributed to the electromer of PVK. This emission is only visible in EL, not in PL, because photoexcitation does not normally generate the free charge carriers needed to form this complex.

For LEDs doped with rGO, the position of the f-PVK around 425 nm was not significantly modified. Additionally, the two shoulders that showed the EL curves of hybrid LEDs did not exhibit variations in wavelength with respect to the PVK-reference. Therefore, the rGO modified the emission intensity of the EL spectra, but this did not change the shape of the EL curves.

To verify that rGO does not modify the electroluminescence spectra, we compared the CIE 1931 chromatic coordinates of the manufactured LEDs. The colour coordinates for PVK-LED were (0.28, 0.28). The use of rGO did not qualitatively modify the chromatic coordinates. These were (0.28, 0.29) for PEDOT:PSS|rGO [30:1] + PVK; (0.27, 0.27) for PEDOT:PSS|rGO [15:1] + PVK and (0.28, 0.27) for PEDOT:PSS|rGO [5:1] + PVK.

### 3.4. Hybrid LEDs with rGO and CdS NCs

**Figure 7:**
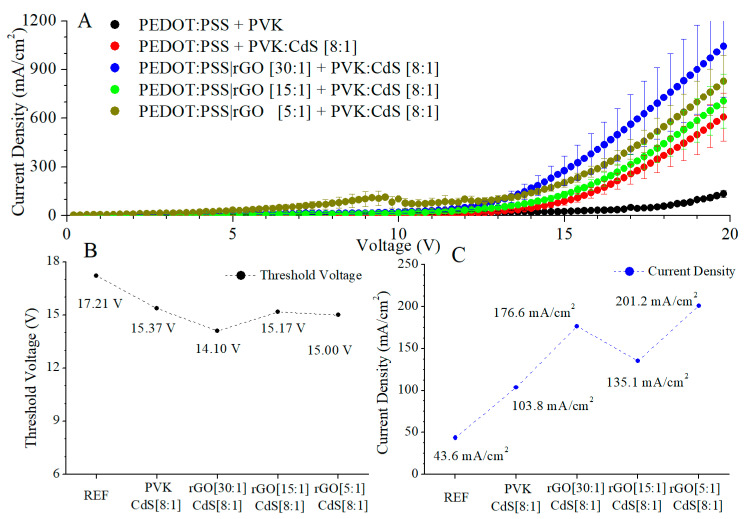
J-V curves (**A**), threshold voltage (**B**), and current density (**C**) at threshold voltage for the hybrid LEDs with rGO in the PEDOT:PSS layer and CdS NCs in the active layer.

Having determined the electrical behaviour due to the inclusion of rGO, we studied the rGO influence on LEDs doped with CdS nanocrystals. In Figure 7A, the J-V curves are plotted. In these curves, it can be observed that at the same voltage values, the current density is higher in the doped LEDs (with any dopant, rGO or CdS NCs) than in the pristine PVK LED. In devices without rGO, the CdS inclusion considerably increased the electrical conduction. Since in PVK LED, the PVK lowest unoccupied molecular orbital (LUMO) is −2.3 eV, and for aluminium it is −4.3 eV, these values cause a potential barrier, the value of which is 2 eV. The inclusion of CdS nanocrystals in the active layer generates distributions where the potential barrier decreases, favouring the electron transport. The conduction band of CdS NCs is closer to that of aluminium than that of PVK LUMO, (the conduction band of CdS NCs is around −4 eV). Therefore, some electrons will occupy the CdS conduction band, producing improvements in electrical behaviour [14]. Additionally, LEDs doped with rGO and their active layer composed of PVK with CdS nanocrystals also increased the electrical conduction of the pristine PVK LED. With the same voltage, the CdS NCs augmented the current density of the LEDs manufactured previously. This modification of electrical behaviour can be justified by changing the hole transport. However, in this case, the improvement of the hole transport is not the only factor present. To the enhancement by rGO is added the influence of the nanoparticles. CdS nanoparticles reduce the potential barrier for electron injection.

In LEDs doped with reduced graphene oxide and CdS nanocrystals, a change was observed. These LEDs had a higher current density at threshold voltage than the reference PVK LED, Figure 7C. Additionally, the doped devices had a lower threshold voltage than the reference, Figure 7B. However, in this case, the direct relation between the rGO load and improvement of electronic transport was eliminated by the presence of the nanoparticles, and was not observed, as indicated in Figure 7B,C. Nevertheless, hybrid LEDs with rGO in the hole transport layer show higher currents and lower threshold voltages than LEDs without rGO. We expected that the electrical conduction would modify with the rGO increase, because more areas of rGO are present, but as shown in Figure 7A, this relation was not produced. The inclusion of CdS nanocrystals produced a higher electrical increase than the inclusion of rGO. In addition, the electrical conduction was higher in LEDs with two dopants than in the devices with a single dopant, but in these devices, the relation “electrical evolution–rGO” was not perceived.

A possible hypothesis to explain this behaviour is related to the morphology of the layers, mainly the interface between the hole transport layer (HTL) and the emissive layer.

Spin-coated layers of PEDOT:PSS are quite smooth, but their roughness increases when rGO is embedded, proportionally to the ratio. If the layer deposited on top of this HTL is pristine polymer, the roughness is corrected to some grade because of the ability of this soft material to coat the underlying surface. Thus, devices with a PVK emissive layer show an influence on the electrical characteristics correlated with the rGO load. On the other hand, if the layer deposited on top of the PEDOT:PSS|rGO layer contains inorganic nanoparticles that are rigid and non-deformable, the interface between the HTL and the emissive layer will have an abrupt profile with several imperfections acting, most probably, as charge carrier traps and recombination centres.

This might be the reason for CdS NCs enriching LEDs with higher rGO load in the HTL; the electrical characteristics do not show the same trend observed for LEDs without CdS nanocrystals.

The electroluminescence of hybrid LEDs doped with rGO and CdS nanocrystals was measured. To observe more clearly the influence of nanoparticles in the EL spectra, the proportion of PVK versus CdS NCs increased to the ratio [PVK:CdS] = [2:1] by weight. Thanks to this increase, the influence of CdS was more evident.

**Figure 8:**
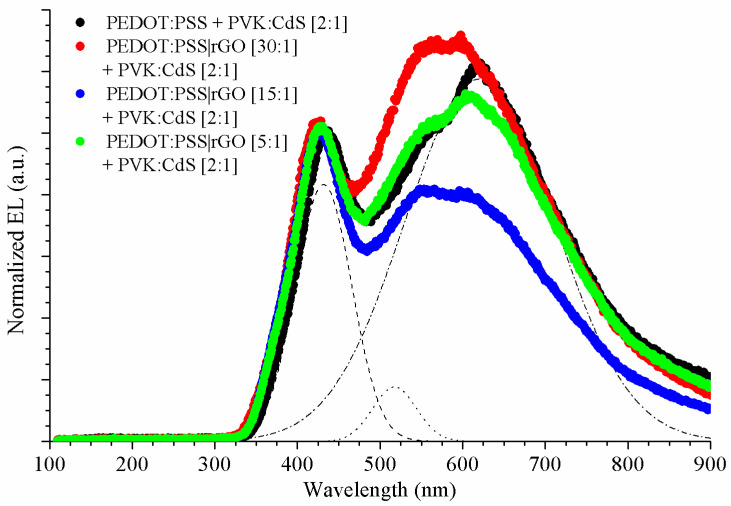
Normalized electroluminescence of PVK LED and hybrid LEDs using rGO in their hole transport layer and doped with CdS NCs. PEDOT:PSS + PVK:CdS [2:1] (black), PEDOT:PSS|rGO [30:1] + PVK:CdS [2:1] (red), PEDOT:PSS|rGO [15:1] + PVK:CdS [2:1] (blue), and PEDOT:PSS|rGO [5:1] + PVK:CdS [2:1] (green). Gaussian deconvolution of PEDOT:PSS + PVK:CdS [2:1] (non-solid lines).

As in devices doped exclusively with rGO, all EL curves of hybrid LED doped with rGO and CdS NCs were normalized, and the average was calculated. All the results are plotted in Figure 8. Gaussian deconvolution was also performed to locate the peak positions of the spectra.

The electroluminescence of hybrid PVK LEDs presented a narrow peak at shorter wavelengths and a broad emission for longer wavelengths. We carried out Gaussian analysis (Table 4) to determine the origin of the broad peak and the composition of the emission spectra.

The narrow peak visible at lower wavelengths (Table 4) is due to the phosphorescence emission of PVK as in PVK-LED reference (428 nm) [14]. The broad peak of hybrid LEDs with CdS NCs was decomposed into two peaks. In Section 3.1, we indicated that two types of nanocrystals were present in the devices: cubic and hexagonal nanocrystals. These different structures are related to different sizes of CdS NCs, so the hybrid LEDs exhibit both contributions in their electroluminescence [14].

The addition of different amounts of rGO to hybrid LEDs with CdS NCs did not significantly shift the wavelength of the emission peaks’ maximum intensity. In Figure 8, the highest peak is identified with CdS nanocrystals. In these devices, the charge carriers from the HOMO of PVK can migrate to the LUMO of PVK or to CdS. Due to this fact, the luminescence of the polymer and the nanoparticles are present in the electroluminescence. In our previous study [14], we demonstrated that with an increasing quantity of nanoparticles, the CdS luminescence is extended, because more charge carriers are able to migrate to CdS. At this point, the quantity of CdS nanoparticles is sufficient for it to account for the highest peak. As in Section 3.3, we studied the CIE 1931 colour coordinates, too. In our previous research, a shift towards the white colour was produced by the inclusion of CdS NCs in pristine PVK-LEDs [14]. The nanocrystals shifted the CIE coordinates to CdS NCs light emission [14]. Thus, we verified the relation of rGO and CdS NCs with PVK-LED, in their chromatic coordinates. The colour coordinates of PEDOT:PSS + PVK:CdS [2:1] were (0.37, 0.34). The nanocrystals produced the abovementioned shift towards the coordinates of CdS light emission (0.45, 0.50). As in the case of the previous study, the presence of rGO did not introduce qualitative variations to the chromatic coordinates of PVK-LEDs doped with CdS NCs. The CIE coordinates of all the manufactured LEDs are collected in Table 5. Apparently, hybrid LEDs with rGO [15:1] and CdS NCs are the closest to D65 white light emission.

## 4. Conclusions

Organic light-emitting diodes with the transport hole layer doped with reduced graphene oxide, and the active layer doped with CdS nanoparticles, were successfully fabricated.

Devices without nanoparticles showed an evolution in their electrical behaviour. This change was proportional to the amount of rGO. The rGO caused a decrease in the threshold voltage and an increase in the current density of the threshold voltage. Devices doped with rGO and CdS modified their electrical behaviour, too. However, the inclusion of CdS nanocrystals eliminated the dependence of the evolution on the amount of rGO.

Electroluminescence measurements were performed. As a result, it was demonstrated that rGO inclusion did not modify the position of the observed emissions in the spectra.

We did not find a clear influence of rGO on the electroluminescent emission in PVK-based LEDs, with or without CdS NCs. The CIE coordinates for LEDs doped with CdS NCs are quite close to white light sources; in particular, the LEDs including an intermediate load of rGO in the PEDOT:PSS layer emitted the light that was closest to the average midday light in Europe, known as D65.

## Figures and Tables

**Table 1:** Summary of the quantities used in the PEDOT:PSS layer doped with rGO.

Solution	PEDOT:PSS (mL)	rGO (mL)
PEDOT:PSS	1.000	0.000
PEDOT:PSS|rGO [30:1]	1.000	0.033
PEDOT:PSS|rGO [15:1]	1.000	0.067
PEDOT:PSS|rGO [5:1]	1.000	0.200

**Table 2:** Summary of the CdS NCs characterization. Absorption edge, PL peak and size.

NCs	Absorption Edge (eV)	Emission Peak (nm)		Size (Brus) (nm)	Size (TEM) (nm)
CdS NCs	3.07	404	576	3.11	3.20 ± 0.06 nm

**Table 3:** Positions of emission peaks for the hybrid LEDs with rGO and PVK LED.

Hybrid LEDs	Gaussian Emission Peaks (nm)		
PEDOT:PSS + PVK	428	495	596
PEDOT:PSS|rGO [30:1] + PVK	427	512	602
PEDOT:PSS|rGO [15:1] + PVK	425	506	594
PEDOT:PSS|rGO [5:1] + PVK	425	505	597

**Table 4:** Positions of emission peaks for the hybrid LEDs with rGO and PVK LED doped with CdS NCs. As reference, pristine PVK LED is also listed.

Hybrid LEDs	Gaussian Emission Peaks (nm)		
PEDOT:PSS + PVK	428	495	596
PEDOT:PSS + PVK:CdS [2:1]	431	517	620
PEDOT:PSS|rGO [30:1] + PVK:CdS [2:1]	417	527	611
PEDOT:PSS|rGO [15:1] + PVK:CdS [2:1]	422	526	613
PEDOT:PSS|rGO [5:1] + PVK:CdS [2:1]	423	521	611

**Table 5:** CIE 1931 colour coordinates of emission peaks for manufactured LEDs.

Hybrid LEDs	CIE 1931 Coordinates
PEDOT:PSS + PVK	(0.28, 0.28)
PEDOT:PSS|rGO [30:1] + PVK	(0.28, 0.29)
PEDOT:PSS|rGO [15:1] + PVK	(0.27, 0.27)
PEDOT:PSS|rGO [5:1] + PVK	(0.28, 0.27)
PEDOT:PSS|rGO [30:1] + PVK:CdS [2:1]	(0.36, 0.37)
PEDOT:PSS|rGO [15:1] + PVK:CdS [2:1]	(0.34, 0.32)
PEDOT:PSS|rGO [5:1] + PVK:CdS [2:1]	(0.36, 0.34)
